# The influence of diabetes and age-related degeneration on body balance control during static standing: a study based on plantar center-of-pressure trajectories and principal component analysis

**DOI:** 10.1186/s13018-023-04129-1

**Published:** 2023-09-30

**Authors:** Xing-xi Hu, Xiong-gang Yang, Xu Wang, Xin Ma, Xiang Geng

**Affiliations:** 1 Department of Orthopedic Surgery, Huashan Hospital, Fudan University, No.12 Wulumuqi Middle Road, Shanghai, 200040 China; 2 Department of Orthopaedics, The First People’s Hospital of Yunnan Province, Affiliated Hospital of Kunming University of Science and Technology, Kunming, 650032 China; 3 Yunnan Key Laboratory of Digital Orthopaedics, Kunming, 650032 China

**Keywords:** Aging, Diabetes mellitus, Balance control, Center of pressure

## Abstract

**Background:**

Aging and diabetes can impair the balance function of the elderly and diabetic patients and increase their fall risk. This study aimed to assess the shaking amplitude of the center-of-pressure (CoP) during static standing, to analyze the effects of aging and diabetes on the balance control.

**Materials and methods:**

This cross-sectional observational study, compared the balance performance of 20 healthy younger adults (27.65 ± 5.60 years), 16 healthy older adults (58.88 ± 3.54 years) and 15 diabetic patients (58.33 ± 5.33 years) in four static standing conditions on a force plate: horizontal, anteroposterior (AP), left and right slope planes (5° angles on AP, left and right directions, respectively). The trajectory coordinates of the CoP over time were recorded and analyzed by principal components analysis to obtain the 95% confidence ellipse and its parameters: angle, major and minor axes lengths, and area. The balance indicators were compared among the three groups using one-way analysis of variance (ANOVA), Brown–Forsythe test or Kruskal–Wallis H test, depending on the normality and homogeneity of variance assumptions.

**Results:**

The diabetic group had a significantly larger confidence ellipse area than the healthy younger adults on the horizontal plane (*P* = 0.032) and than the healthy older adults on the horizontal (*P* = 0.036), AP slope (*P* = 0.023), and right ML slope (*P* = 0.037) planes. There were no significant differences in the major axis length of the confidence ellipse among the three groups. The diabetic group had a significantly longer minor axis length than the healthy younger adults on the AP slope (*P* = 0.039), left ML slope (*P* = 0.045) and right ML slope (*P* = 0.016) planes and than the healthy older adults on the AP slope (*P* = 0.007), left ML slope (*P* = 0.035) and right ML slope (*P* = 0.012) planes.

**Conclusions:**

The balance control of diabetic patients is decreased compared with healthy younger and older people, and the body swing amplitude increases mainly in the direction of minor axis of confidence ellipse during static standing, while the swing amplitude in the direction of the major axis has no significant change. Evaluating the balance function of diabetic patients can help clinicians identify people with fall risk early and intervene early, thereby reducing the occurrence of fall events in this population.

## Introduction

Falls are a common and serious problem for older adults and people with diabetes. About 39% of people over 65 years old fall each year [1], and this risk is more than three times higher for those with poor glycaemic control [2]. Falls can cause severe injuries, disability, and even death, affecting the mobility and quality of life of the elderly or diabetic population [3]. In 2016, one older adult was admitted to the emergency room every 11 s because of a fall, and one died every 19 min [4]. In the United States, 29 million older adults fell in 2014, and this number is projected to increase to 74 million by 2030 [4]. Balance impairment is the main risk factor for falls in the elderly and diabetic population [5]. Silva R et al. [6] reported that exercise prevents falls by improving balance and strength. Different types of exercise have different effects on the rate of falls, which can be reduced by 23–42%, A community program can encourage older women to exercise. Balance is a complex skill that involves multiple motor nerves, muscles, and cognitive processes [7]. It depends on three sensory systems: the somatosensory system, which provides information on body position and movement; the visual system, which provides information on the environment and orientation; and the vestibular system, which provides information on head position and spatial orientation [8]. Any impairment in these sensory systems can reduce balance and increase the risk of falls [7]. In people with diabetes, diabetic peripheral neuropathy (DPN) is the primary cause of somatosensory impairment [9]. Diabetic retinopathy can also impair vision and balance in diabetic patients. Moreover, some studies have reported that diabetic patients without DPN may also have reduced somatosensory function and balance [10–12]. In older adults without diabetes, aging can also affect the somatosensory, visual, and vestibular systems, leading to balance decline. The study of aged falling is important because it can help prevent injuries, improve quality of life, and reduce health care costs for older adults. According to Irandoust et al. [13], yoga and pilates exercises can enhance the balance, flexibility, strength, and endurance of older adults, which can reduce the risk of falling. Similarly, Seghatoleslami et al. [14] found that pilates exercises can improve the motor control of inactive middle-aged women, which can also prevent falls. Therefore, the study of aged falling can contribute to the promotion of physical and mental health for the aging population.

Gait changes, such as reduced walking speed, increased stride variability, and increased plantar stress, are also associated with increased fall risk in diabetic patients [15]. Moreover, psychological factors, such as fear of falling and balance confidence, can affect the daily activities and social behavior of older people, leading to physical decline, depression, social isolation, and helplessness [16, 17]. A systematic review by Hewston et al. [17] found that psychological factors may have a greater impact on falls in type II diabetes than DPN and other physical factors. Therefore, early identification and intervention for people with balance dysfunction can help to reduce the fear of falling and improve balance confidence. For example, gait and balance training, Tai Chi, or yoga may be beneficial [17]. Another study by Mickle et al. [18] found that foot pain and increased plantar stress were more common in older adults with a history of falls than those without. This suggests that foot pain and plantar stress may also contribute to the fall risk in the elderly and should be addressed accordingly [18].

Assessing balance in the elderly and diabetic population is important to improve their balance confidence and reduce their fall risk [19–25]. The main assessment methods include: (1) fall risk assessment scales, which use various tools to measure the fear of falling and balance confidence in older people and diabetics. However, these scales are not very accurate in predicting the fall risk, so a combination of two or more scales is recommended [20]. (2) Functional performance tests, which use simple balance tasks, such as standing on one or two legs or walking on a balance beam, to score the balance ability. These methods are easy to use but subjective and prone to errors. (3) Center of mass (CoM) position measurement, which uses optical or inertial sensors to record the changes in the CoM position during human movement, and then calculates the direction and rate of CoM movement to reflect the balance function. This method is more accurate and objective but also complex, expensive, and time-consuming. (4) Center of pressure (CoP) measurement, which has become the “gold standard” in balance assessment [26]. It uses a force plate to record the CoP trajectory and plantar pressure distribution during standing. A study by Fan et al. [27] found that patients with diabetic foot ulcers (DFU) had higher CoP ellipse area, CoP trajectory length, and CoP trajectory angle than healthy adults, indicating impaired balance. They also found that plantar pressure was unevenly distributed between the affected and healthy sides in DFU patients. Assessing balance in different slope orientations is important for preventing falls in this population, as they may encounter complex road conditions in their daily activities, such as stairs or inclined surfaces, which increase the fall risk. In this study, we used a force plate to assess the static balance of the elderly and diabetic population in different standing phases (horizontal plane, anteroposterior (AP) slope plane, left slope plane and right slope plane), to analyze the effects of aging and diabetes on balance.

## Materials and methods

This cross-sectional observational study was conducted at Huashan Hospital affiliated to Fudan University. The following three groups of subjects were included: (1) Healthy younger group: aged between 18 and 40 years without a history of diabetes. (2) Healthy older group: aged between 50 and 70 years without a history of diabetes. (3) Diabetic group: adults with a definitive diagnosis of type II diabetes based on the diagnostic criteria in the Chinese Guidelines for the Prevention and Treatment of Type 2 Diabetes (2020 Edition) [28].

### Inclusion and exclusion criteria

Patients were included according to the following criteria: (1) adults aged between 18 and 70 years; (2) able to complete a normal gait cycle autonomously and cooperate with physical examination and relevant tests; (3) no lower limb-related disorders other than diabetes that may affect plantar mechanics; (4) diabetic patients without active ulcer and ulcer history.

Exclusion criteria: (1) lower limb-related conditions such as ankle deformity, heel pain, stroke, and knee/ ankle arthritis that may affect gait; (2) foot ulcers.

Subjects were enrolled in strict compliance with the Declaration of Helsinki and approved by Ethics Committee of the Huashan Hospital Affiliated to Fudan University, and all subjects were enrolled with informed consent.

### Data acquisition

**Fig. 1:**
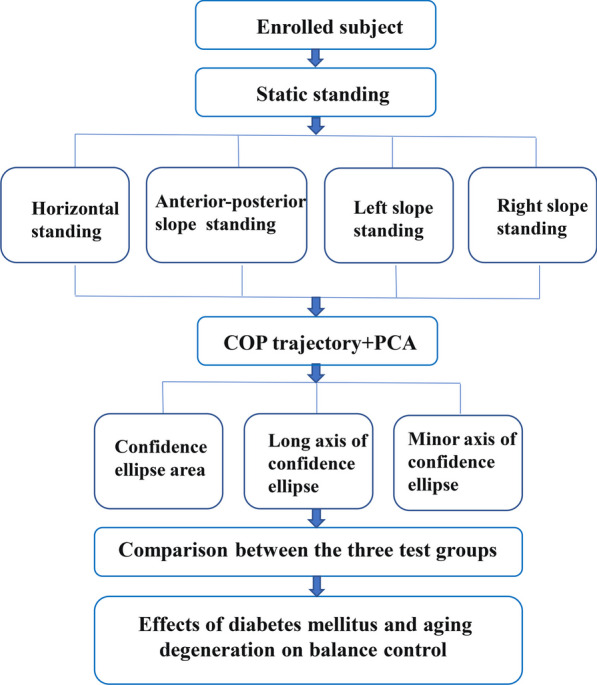
A flowchart for balance test based on plantar force plate and the centre of pressure (CoP) recording. Principal component analysis (PCA) was performed on the CoP trajectories, and 95% confidence ellipses were drawn, to calculate the area, long axis length, short axis length and major axis direction of confidence ellipses

The flowchart of the study is shown in Fig. 1. The subject fully exposes the lower leg and ankle, stands statically on force plate, a custom-built force platform, consisting of a pressure mat with 2400 sensing elements mounted flush onto a high-precision six-axis force/moment plate (600 × 400 mm^2^), and the information on the accuracy and precision of force plate and pressure mat measurements was detailed by Qian et al. [29]. Subjects were instructed to stand upright on the platform’s central area and stand as immobile as possible for a period of 30 s, and collects the coordinates of CoP trajectory over time. The subjects completed the following four balance tests in static standing on the force plate: (1) horizontal plane: the force plate was placed horizontally on the ground; (2) AP slope plane: an AP angle of 5° between force plate and horizontal plane was created; (3) left slope plane: an (ML) angle of 5° on the left side between force plate and horizontal plane was created; (4) right slope plane: an ML angle of 5° on the right side between force plate and horizontal plane was created. The angle between force plate and horizontal plane was adjusted by six degrees of freedom disturbance platform, Customised WIN06-010A standard model by Shanghai Yinghao Mechanical & Electrical Equipment Co.

The above four steps were repeated three times and the average of the three experiments was taken as the final experimental result. CoP-related data acquisition is done at a frame rate of 100 frames per second. As the human body’s balance ability is affected by many factors such as physical health, physiology, and psychology, the test environment was chosen to be quiet and comfortable, with a room temperature of around 25 °C, to avoid the influence of the surrounding environment and ambient temperature on the static stability of the subject.

### Data processing

**Fig. 2:**
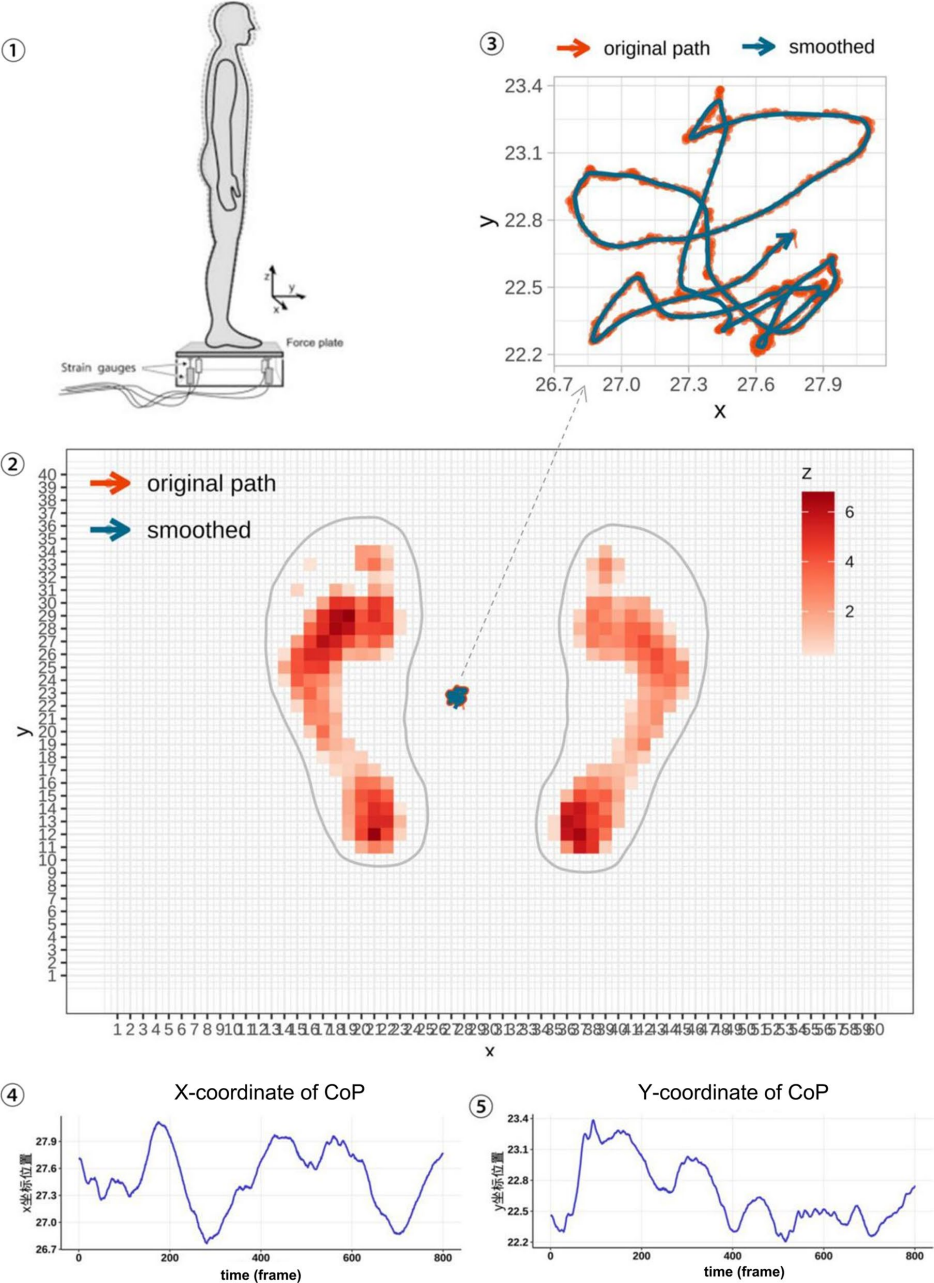
The process of collecting center of pressure (CoP) data and extracting CoP coordinates based on the plantar force plate. ① The test was conducted with the human body standing statically on the force plate and maintaining rest for 30 s, and the plantar pressure distribution data was recorded; ② The plantar pressure distribution map was plotted and the x and y coordinates of the CoP were calculated for each frames; ③ CoP trajectory plotted using the x and y coordinates of CoP; ④ Curve of time and CoP-x coordinate; ⑤ Curve of time and CoP-y coordinate

The data collection of plantar pressure distribution on a subject during static standing, and the COP trajectory extraction steps are shown in Fig. 2. Assuming that the pressure p is distributed over a plane of area S located within the Oxy plane, that the direction of action of p is negative in the z-axis direction, and that p is set to be a function of x and y only, the pressure dF acting on a unit area dS is:

$${\text{dF }} = {\text{ p}}\left( {{\text{x}},{\text{ y}}} \right) \cdot {\text{dS }} = {\text{ p}}\left( {{\text{x}},{\text{ y}}} \right) \cdot {\text{d}}_{{\text{x}}} \cdot {\text{d}}_{{\text{y}}}$$

The resultant force of the pressure acting on the entire plane is:

$${\text{F }} = \int\limits_{{\text{S}}} {{\text{p}}\left( {{\text{x}},{\text{ y}}} \right) \cdot {\text{d}}_{{\text{x}}} \cdot {\text{d}}_{{\text{y}}} } { }$$

Therefore, the coordinate of pressure center (COP) (xc,yc) is:

$${\text{x}}_{{\text{c}}} { } = { }\frac{{\smallint_{{\text{s}}} {\text{ x}} \cdot {\text{p}}\left( {{\text{x}},{\text{ y}}} \right) \cdot {\text{d}}_{{\text{x}}} \cdot {\text{d}}_{{\text{y}}} }}{{\smallint_{{\text{s}}} {\text{ p}}\left( {{\text{x}},{\text{ y}}} \right) \cdot {\text{d}}_{{\text{x}}} \cdot {\text{d}}_{{\text{y}}} }}$$

$${\text{y}}_{{\text{c}}} { } = { }\frac{{\smallint_{{\text{s}}} {\text{ y}} \cdot {\text{p}}\left( {{\text{x}},{\text{ y}}} \right) \cdot {\text{d}}_{{\text{x}}} \cdot {\text{d}}_{{\text{y}}} }}{{\smallint_{{\text{s}}} {\text{ p}}\left( {{\text{x}},{\text{ y}}} \right) \cdot {\text{d}}_{{\text{x}}} \cdot {\text{d}}_{{\text{y}}} }}$$

**Fig. 3:**
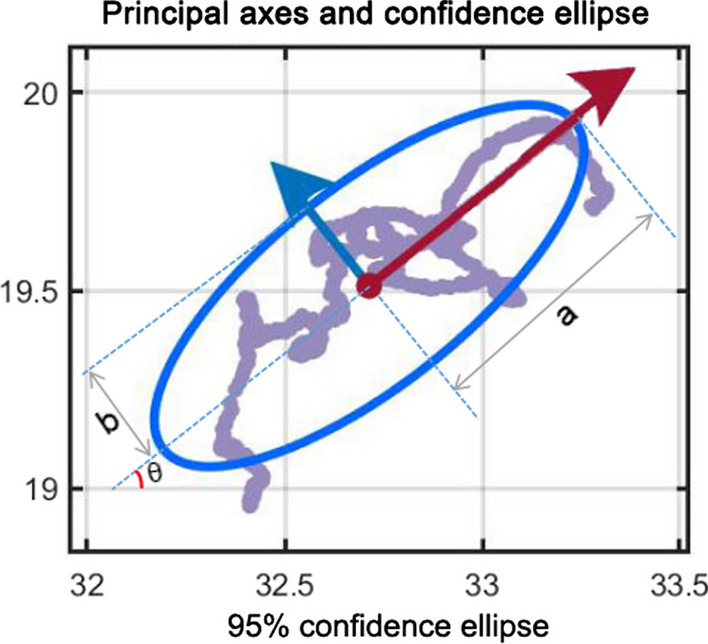
The 95% confidence ellipse for the center of pressure (CoP) trajectory using principal component analysis. The blue ellipse is the 95% confidence ellipse, and the red and blue arrows are the major (or long) and minor (or short) axes of the confidence ellipse respectively. In the figure, “a” is the length of the long axis of the confidence ellipse (long semi-axis), “b” is the length of the short axis of the confidence ellipse (short semi-axis), and the area of the confidence ellipse is “S = π·a·b”. The angle θ is the angle between the principal axis of the confidence ellipse and the x-axis

The plantar pressure nephogram is plotted and the x and y coordinates of the CoP are calculated for each frame according to the above algorithm, and the CoP trajectory is plotted on the basis of the plantar pressure nephogram. PCA is a statistical method that reduces the dimensionality and extracts the features of multiple correlated variables by transforming them into a few uncorrelated variables, called principal components, that preserve most of the original data information. PCA can process the data of pressure center measurement, to identify the main features of the pressure center trajectory, such as direction, shape, size, etc., and to remove noise and redundant data. The obtained CoP trajectories were further analyzed by PCA using Matlab R2021a software (MathWorks, Massachusetts, USA) to extract the principal axes of the CoP trajectories and draw 95% confidence ellipses, reflecting the magnitude of sway in the main sway direction (a) and the direction perpendicular to the main sway direction (b), respectively, and the area of the 95% confidence ellipse (S = π·a·b), reflecting the overall magnitude of human sway (Fig. 3).

### Statistical analysis

The balance parameters obtained under different test conditions are described in the form of “mean ± standard deviation (SD)”. When comparing baseline information among the three groups: Pearson’s chi-square test or Fisher’s exact probability method was chosen accordingly, based on expected frequency. When comparing continuous baseline data and balance indexes among the three groups: (1) if all three groups were normally distributed and met the assumption of homogeneity of variance (HoV), the ANOVA was used for comparison, and the SNK-q test was used for post hoc comparisons; (2) if all three data sets were normally distributed but did not follow the assumption of HoV, the Brown–Forsythe test was used for comparison, and the Tamhane’s T_2_ test was used for post hoc comparisons; (3) if the data did not follow a normal distribution, the non-parametric Kruskal–Wallis H test was chosen, and the Dunnett’s test was used for post hoc comparisons. The balance parameters obtained for the three groups under different test conditions were plotted as cloud-rain scatter plots to characterize the distribution of the data. Nuclear density maps and circular radiation plots were plotted for the angle between the principal axis of the confidence ellipse and the x-axis to characterize the distribution in the principal axis direction. To assess the degree of concentration of the distribution of the angle between the principal axis direction and the x-axis among three subject groups, the kurtosis coefficients were calculated separately. The kurtosis of a perfectly normal distribution is 0. If the kurtosis is greater than 0, the distribution is more concentrated (or steeper) than normal, and if the kurtosis is less than 0, the distribution is more dispersed (or fatter) than normal, so overall the larger the kurtosis the more concentrated the distribution. Normality and HoV tests were performed using the Shapiro–Wilk test (W test, *P* < 0.05 means the data do not follow a normal distribution) and the modified Bartlett’s test (*P* < 0.05 means the data do not meet the assumption of HoV test), respectively.

The above data analyses were done using R language version 4.2.2 (Foundation for Statistical Computing, Vienna, Austria). A two-sided *P* < 0.05 was taken as a statistically significant difference.

## Results

### Demographic of the enrolled population

The demographic information of the three groups is shown in Table 1. Twenty (40 feet), 16 (32 feet) and 15 (30 feet) subjects were enrolled in healthy younger, healthy older and diabetic groups respectively. There was no significant difference among the three groups in terms of gender distribution, but mean age was significantly higher in the healthy older and diabetic groups than in the healthy younger group. There was no significant difference on age between the healthy older and diabetic groups. A significantly higher BMI was found in diabetic group than healthy younger adults, and no significant difference in incidence of plantar callus was found among the three groups.

**Table 1:** Comparison results of demographic of three groups of subjects

Demographic	Group			Comparison among three groups	A versus B	A versus C	B versus C
	Healthy younger group (A) (20cases/40 foot)	Healthy older group (B) (16 cases/32 foot)	Diabetic group (C) (15 cases/30 foot)				
*Sex*							
Male	11 (55.0%)	7 (43.75%)	7 (46.67%)	Χ^2^ = 0.497; *P* = 0.780	Χ^2^ = 0.450; *P* = 0.502	Χ^2^ = 0.238; *P* = 0.625	Χ^2^ = 0.027; *P* = 0.870
Female	9 (45.0%)	9 (56.25%)	8 (53.33%)				
*Age (year)*	27.65 ± 5.60	58.88 ± 3.54	58.33 ± 5.33	F = 236.765; *P* < 0.001***	MD = − 31.23; *P* < 0.001***	MD = − 30.68; *P* < 0.001***	MD = 0.54; *P* = 0.754
*BMI (kg/m*^*2*^*)*	22.01 ± 2.18	23.31 ± 2.35	24.88 ± 4.43	H = 7.939; *P* = 0.019*	D = 1.607; *P* = 0.186	D = 2.741; *P* = 0.012*	D = 0.689; *P* = 0.710
*Smoking*							
Yes	6 (30.00%)	6 (37.50%)	5 (33.33%)	Χ^2^ = 0.225; *P* = 0.894	Χ^2^ = 0.226; *P* = 0.635	Χ^2^ = 0.044; *P* = 0.833	Χ^2^ = 0.059; *P* = 0.809
No	14 (70.00%)	10 (62.50%)	10 (66.67%)				
*Drinking*							
Yes	6 (30.00%)	5 (31.25%)	3 (20.00%)	Fisher’s; *P* = 0.789	Fisher’s; *P* = 1.000	Fisher’s; *P* = 0.700	Fisher’s; *P* = 0.685
No	14 (70.00%)	11 (68.75%)	12 (80.00%)				
*Hypertension*							
Yes	2 (10.00%)	8 (50.00%)	10 (66.67%)	Χ^2^ = 12.684; *P* = 0.002**	Fisher’s; *P* = 0.011*	Χ^2^ = 12.216; *P* < 0.001***	Χ^2^ = 0.883; *P* = 0.347
No	18 (90.00%)	8 (50.00%)	5 (33.33%)				
*Coronary heart disease*							
Yes	0 (0.00%)	0 (0.00%)	1 (6.67%)	Fisher’s; *P* = 0.294	NA	Fisher’s; *P* = 0.429	Fisher’s; *P* = 0.484
No	20 (100.00%)	16 (100.00%)	14 (93.33%)				
*Plantar callus*							
Yes	8 (40.00%)	4 (25.00%)	8 (53.33%)	Χ^2^ = 2.616; *P* = 0.270	Χ^2^ = 0.900; *P* = 0.343	Χ^2^ = 0.614; *P* = 0.433	Χ^2^ = 2.620; *P* = 0.106
No	12 (60.00%)	12 (75.00%)	7 (46.67%)				
Average	2.13 ± 0.99	2.25 ± 1.26	2.63 ± 0.92	H = 2.217; *P* = 0.330	D = 0.250; *P* = 0.958	D = 1.511; *P* = 0.234	D = 0.622; *P* = 0.692
*Heel callus*							
Yes	2 (10.00%)	0 (0.00%)	1 (6.67%)	Fisher’s; *P* = 0.624	Fisher’s; *P* = 0.492	Fisher’s; *P* = 1.000	Fisher’s; *P* = 0.484
No	18(90.00%)	16(100.00%)	14(93.33%)				

Χ^2^, Pearson’s Chi square test; F, univariate Analysis of Variance; H, Kruskal–Wallis H test; D, the effect size of Dunnett's test; MD(mean difference), the mean of the difference between two groups of continuous variables; Fisher’s exact probability method is used as an alternative when Pearson’s Chi-square test is not satisfied. *P < 0.05, **P < 0.01, ***P < 0.001

### Influence of diabetes and age-related degeneration on human balance

**Fig. 4:**
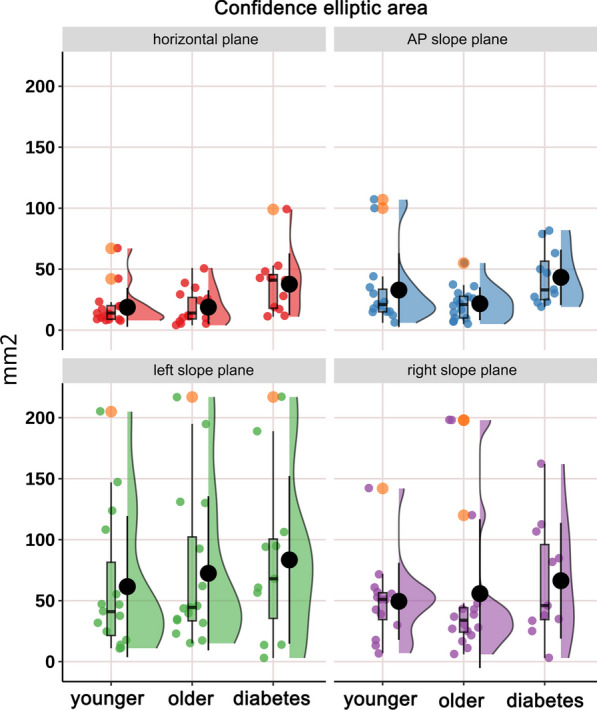
Cloud-rain plots for comparisons of the center of pressure (CoP) confidence ellipse area for healthy young people, healthy older people, and diabetic patients in static stance (horizontal, anterior–posterior slope, left slope, and right slope stances). The clouds in the graph represent the kernel density distribution of the data, the rain dots represent each sample, the orange circles represent the outliers, the black dots and error lines represent the means and standard deviations, and the box plots represent the medians and their quartiles

**Fig. 5:**
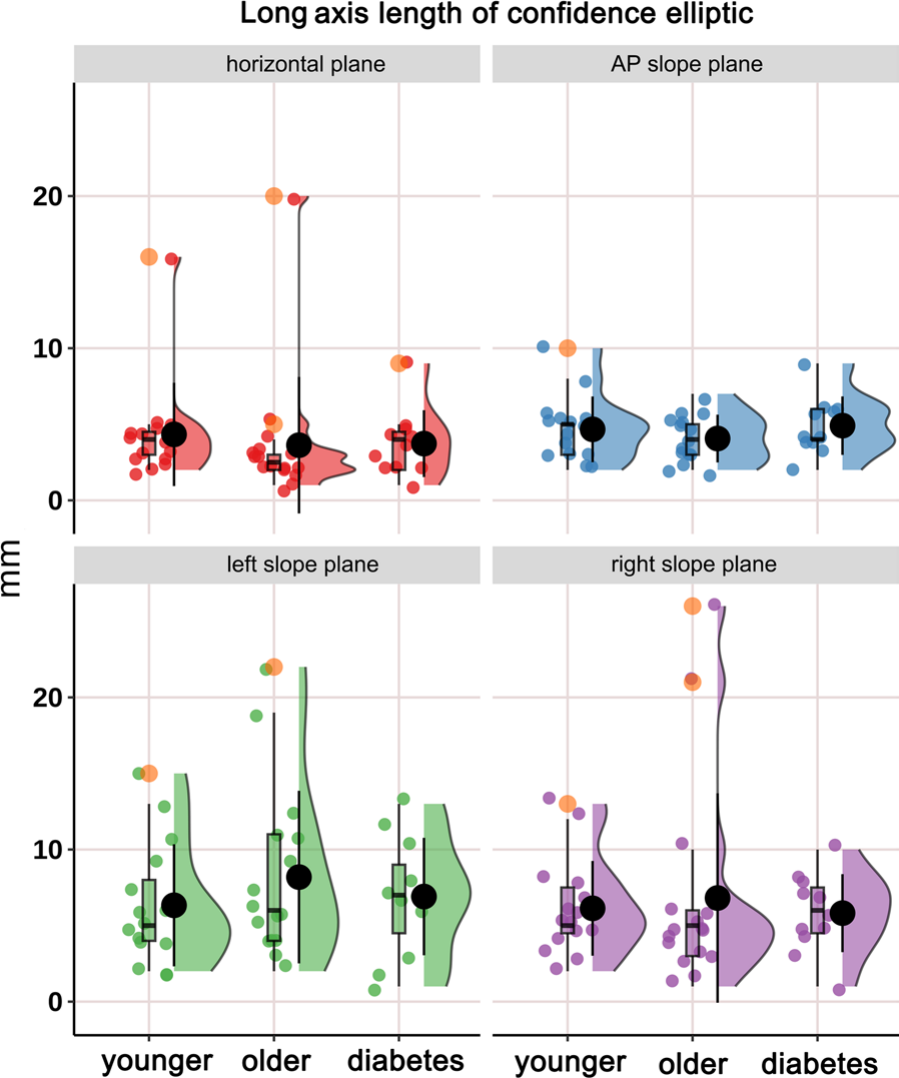
Cloud-rain plots comparing the length of the long axis of the center of pressure (CoP) confidence ellipse for healthy younger people, healthy older people and diabetic patients in static stance (horizontal, anterior–posterior slope, left slope and right slope stances). The clouds in the graph represent the kernel density distribution of the data, the rain dots represent each sample, the orange circles represent the outliers, the black dots and error lines represent the means and standard deviations, and the box plots represent the medians and their quartiles

**Fig. 6:**
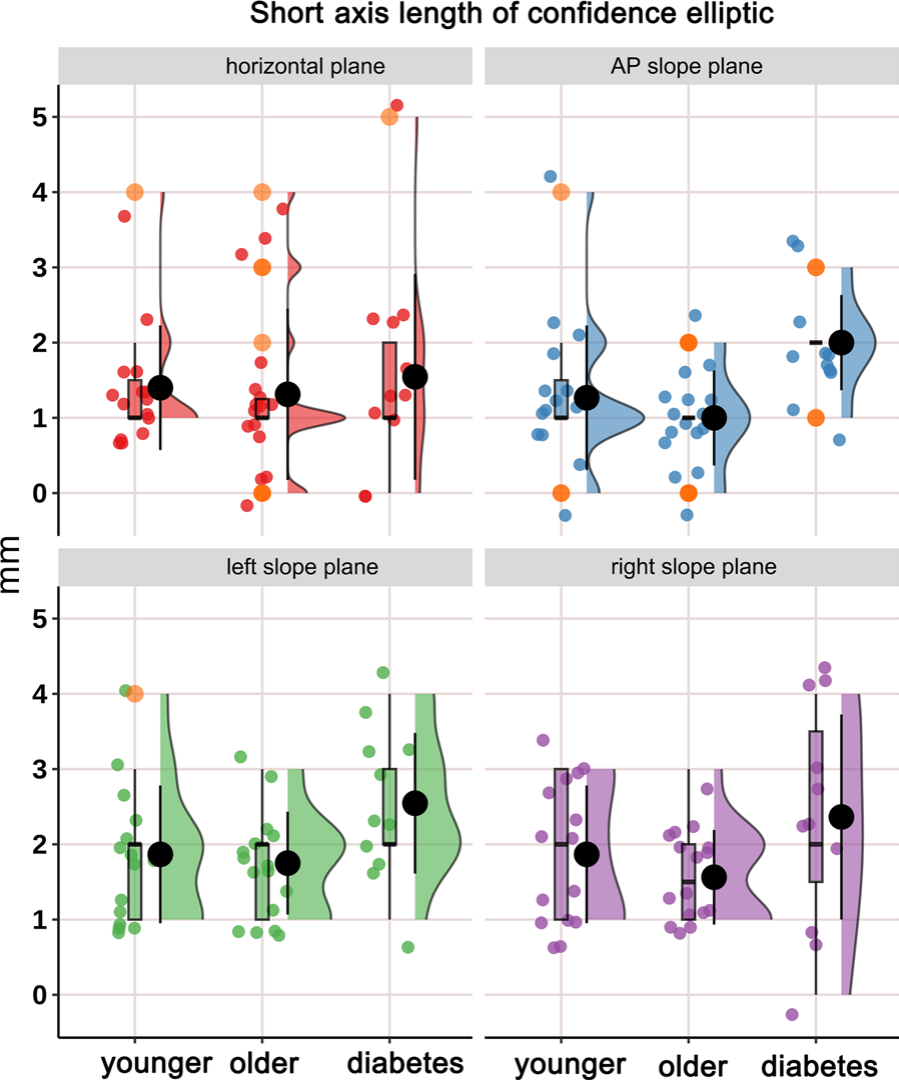
Cloud-rain plots comparing the length of the short axis of the center of pressure (CoP) confidence ellipse for healthy younger people, healthy older people and diabetic patients in static stance (horizontal, anterior–posterior slope, left slope and right slope stances). The clouds in the graph represent the kernel density distribution of the data, the rain dots represent each sample, the orange circles represent the outliers, the black dots and error lines represent the means and standard deviations, and the box plots represent the medians and their quartiles

The distribution of CoP confidence ellipse area, long axis length and short axis length in static stance (horizontal stance, AP slope stance, left slope stance and right slope stance) in healthy younger people, healthy older people and diabetic patients and the differences among the groups are shown in Figs. 4, 5 and 6 and Tables 2, 3 and 4.

**Fig. 7:**
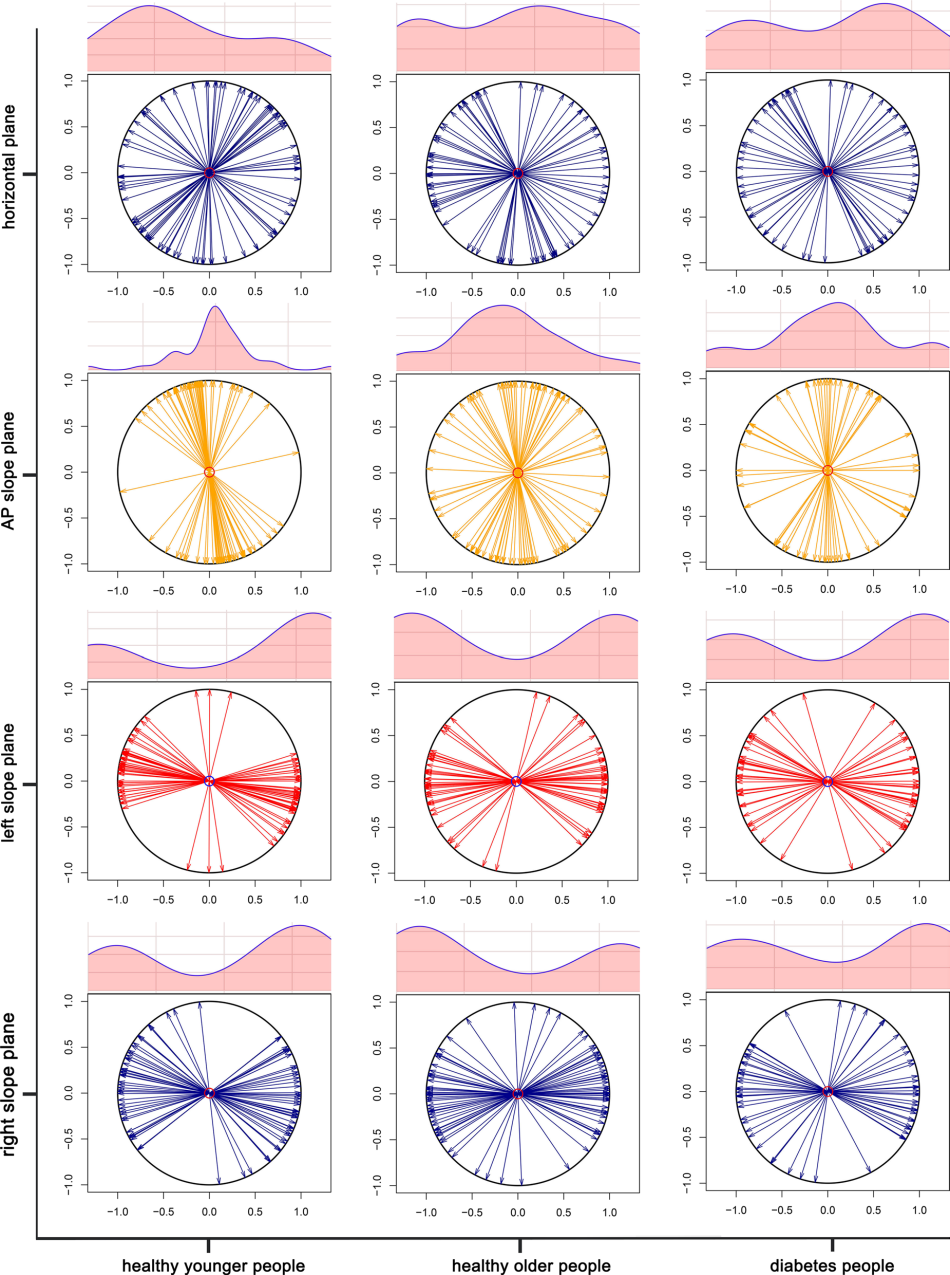
Nuclear density and circular radiation plots of the principal axis direction distribution of the center of pressure (CoP) confidence ellipse for the three groups in static stances (horizontal, anterior–posterior slope, left slope, and right slope stances). The nuclear density plot represents the distribution in the principal axis direction between 0° and 180°, and the arrows in the circular radiation plot represent the principal axis direction of the CoP confidence ellipse (i.e. the direction of the subject's main sway when standing) at each test. As can be seen, the sway direction is relatively evenly distributed across all angles when standing horizontally, with no significant differences between the three groups. The direction of sway was more concentrated in the anterior–posterior and left–right directions when standing on the anterior–posterior and left–right slopes, respectively, and the main axis of sway was more concentrated in healthy younger subjects than in healthy older, and diabetic subjects

1. Confidence ellipse area (Table 2 and Fig. 4): there was a significant difference in the CoP confidence ellipse area among the three groups in static horizontal stance (H-test, *P* = 0.023*), anterior–posterior slope stance (H-test, *P* = 0.039*) and right-side slope stance (H-test, *P* = 0.028*). Post hoc comparisons revealed that: (i) the diabetes group had a significantly higher confidence ellipse area in static horizontal stance (*P* = 0.032*) relative to healthy younger adults; (ii) the diabetes group had significantly higher confidence ellipse areas for static horizontal stance (*P* = 0.036*), AP slope stance (*P* = 0.023*), and right-side slope stance (*P* = 0.037*) relative to healthy older adults.
2. Long axis length of confidence ellipse (Table 3 and Fig. 5): there was no significant difference in the amplitude of sway in the long axis direction of the confidence ellipse among the three groups in the overall and any pairwise comparison.
3. Short axis length of confidence ellipse (Table 4 and Fig. 6): the length of the short axis of the confidence ellipse during static AP slope stance (H-test, *P* = 0.006**), left slope stance (one-way ANOVA, *P* = 0.021*), and right slope stance (one-way ANOVA, *P* = 0.014*) were significantly different overall among the three groups. The post hoc analyses showed that: (i) Compared with the healthy younger adults, the length of short axis of the confidence ellipse increased significantly in the diabetic group during static AP slope standing (*P* = 0.039*), left slope standing (0.045*) and right slope standing (0.016*); (ii) Compared with the healthy older group, the short axis length of confidence ellipse was significantly increased in the diabetic group during static AP slope standing (*P* = 0.007**), left slope standing (*P* = 0.035*) and right slope standing (*P* = 0.012*).
4. Major axis orientations of confidence ellipse: Fig. 7 shows the frequency distribution density and circular radiation plot depicting the direction of main axis of confidence ellipse for the three groups in static stance (horizontal plane, AP slope, left slope and right slope). In the horizontal stance, the sway directions were relatively evenly distributed across the angles, with no significant differences in the distribution trends among three groups. On the AP, left, and right slopes, the direction of sway was concentrated in the AP, left and right directions respectively, and the major axis of sway was more concentrated in one direction in the healthy younger subjects than in the healthy older and diabetic subjects. The nuclear density map also shows that in the AP static slope stance, the major axis direction is more concentrated around 90° and more concentrated in the healthy younger people than in the other two groups, whereas, in left and right static-slope stances, the kernel density map is mainly concentrated near 0° and 180°). Again the younger subjects showed a evident trend toward concentration compared to the other two groups. Table 5 shows the kurtosis coefficients for the distribution of the angles between the major axis of the CoP confidence ellipse and the x-axis. The kurtosis coefficients for the healthy younger group were optimal for all four test conditions and were significantly better than the other two groups in AP and ML slope stances, indicating that healthy older people and diabetic patients have a greater degree of instability in the direction perpendicular to the direction of major axis of confidence ellipse than healthy younger subjects.

**Table 2:** Confidence elliptic area of three groups of subjects during static standing

Test condition	Group A (mm^2^)	Group B (mm^2^)	Group C (mm^2^)	*P* (overall)	*P* (A–B)	*P* (A–C)	*P* (B–C)
Horizontal	19.18 ± 16.10	19.40 ± 13.80	37.95 ± 25.33	0.023^H*^	0.996	0.032*	0.036*
Anterior–posterior slope	33.27 ± 30.27	22.25 ± 13.53	43.82 ± 22.89	0.039^H*^	0.652	0.155	0.023*
Left slope	61.98 ± 57.76	72.97 ± 63.06	83.83 ± 68.69	0.651^H^	0.776	0.673	0.922
Right slope	50.04 ± 31.46	42.46 ± 49.06	66.15 ± 35.71	0.028^H*^	0.135	0.761	0.037*

Mean areas and analysis of variance results for the center of pressure (CoP) confidence ellipse in static standing (horizontal, anterior–posterior slope, left slope, and right slope standing) for healthy younger (group A), healthy older ((group B) and diabetic (group C) subjects. H represent effect size from Kruskal–Wallis H-test, and correspondingly post hoc multiple comparisons were performed using Dunnett's test. *P < 0.05

**Table 3:** Long axis length of confidence ellipse among three groups during static standing

Test condition	Group A (mm)	Group B (mm)	Group C (mm)	*P* (overall)	*P* (A–B)	*P* (A–C)	*P* (B–C)
Horizontal	4.66 ± 3.34	4.19 ± 4.58	4.28 ± 2.14	0.184^H^	0.204	1.000	0.326
Anterior–posterior slope	5.22 ± 2.15	4.42 ± 1.60	5.33 ± 1.83	0.365^F^	0.267	0.887	0.420
Left slope	6.75 ± 3.97	8.86 ± 5.63	7.41 ± 3.86	0.558^H^	0.506	0.707	0.977
Right slope	6.73 ± 3.12	7.36 ± 6.84	6.38 ± 2.55	0.522^H^	0.530	1.000	0.607

Mean long-axis lengths and analysis of variance results for the center of pressure (CoP) confidence ellipse in static standing (horizontal, anterior–posterior slope, left slope, and right slope standing) for healthy younger (group A), healthy older ((group B) and diabetic (group C) subjects. F and H represent effect sizes from one-way ANOVA and Kruskal–Wallis H-test, and correspondingly post hoc multiple comparisons were performed using the SNK-q test and Dunnett's test, respectively

**Table 4:** Short axis length of confidence ellipse among three groups during static standing

Test condition	Group A (mm)	Group B (mm)	Group C (mm)	*P* (overall)	*P* (A–B)	*P* (A–C)	*P* (B–C)
Horizontal	1.88 ± 0.88	2.10 ± 0.68	2.03 ± 1.26	0.569H	0.498	0.959	0.788
Anterior–posterior slope	1.75 ± 0.85	1.54 ± 0.53	2.33 ± 0.49	0.006H**	0.812	0.039*	0.007**
Left slope	2.29 ± 0.81	2.27 ± 0.69	3.09 ± 0.90	0.021F*	1.000	0.045*	0.035*
Right slope	2.28 ± 0.88	2.10 ± 0.68	3.13 ± 1.14	0.014F*	0.606	0.016*	0.012*

Mean short-axis lengths and analysis of variance results for the center of pressure (CoP) confidence ellipse in static standing (horizontal, anterior–posterior slope, left slope, and right slope standing) for healthy younger (group A), healthy older ((group B) and diabetic (group C) subjects. F and H represent effect sizes from one-way ANOVA and Kruskal–Wallis H-test, and correspondingly post hoc multiple comparisons were performed using the SNK-q test and Dunnett’s test, respectively. *P < 0.05, **P < 0.01

**Table 5:** Kurtosis coefficient of CoP confidence Angle distribution between elliptic spindle and X-axis of subjects in three groups during static standing

Test condition	healthy young people	healthy middle-aged	diabetic patients
Horizontal	− 1.010	− 1.323	− 1.445
Anterior–posterior slope	2.607	− 0.217	− 0.578
Left slope	4.707	0.719	− 0.046
Right slope	1.443	− 0.602	− 0.338

The kurtosis coefficient is used to assess the degree of concentration (or steepness) of the distribution of the angle between the principal axes of the CoP confidence ellipse and the x-axis. The kurtosis value of a perfectly normal distribution is equal to 0. When the kurtosis value is > 0, it means that the data distribution is relatively high compared to the normal distribution, and when the kurtosis value is < 0, it means that the data distribution is relatively short compared to the normal distribution, so the larger the kurtosis value is, the more concentrated the distribution of the angle between the main axis direction and the x-axis

## Discussion

A possible explanation for the discrepancy between our results and those of other studies [6] that reported a larger CoP sway in the direction of the major axis of the confidence ellipse is that we used different standing conditions and force plate orientations. Our findings suggest that diabetes and age-related degeneration have different effects on the balance control during static standing, which is in contrast to other studies that assumed a similar mechanism of balance impairment in both conditions. For older people and diabetes patients, the key to preventing falls is to identify those at risk of falling early and to manage them with appropriate interventions. To do this requires an adequate assessment of the factors that may contribute to an increased risk of falls. Akbari et al. [30] suggested that micronutrient intake can modulate the oxidative stress, inflammation, and neurogenesis in the brain, which can influence the cognitive performance of the elderly. Vancini et al. [31] proposed that ultra-endurance exercises can induce neuroplasticity, neurogenesis, and angiogenesis in the brain, which can enhance the cognitive function and physical activity of the elderly. However, while the serious consequences of falls and the associated high healthcare costs are well recognized, there is still a lack of key sensitivities that can effectively screen people at risk of falls. Over 400 factors have been reported to be associated with the risk of falls in the adult population [32]. The psychological fear of walking (i.e. ‘fear of falling’) is also being recognized as a very important health issue and has been the subject of considerable research in recent years [16, 17, 20]. Data report that nearly 13 million (36%) older Americans (more than 65 years) have a moderate or severe fear of falling and that there is a strong correlation between the fear of experiencing a fall and the actual occurrence of a fall [33]. To assess the risk of falls in the elderly and diabetic populations, several scales have been designed to predict the future risk of falls in these populations from different perspectives [20, 34, 35]. However, these scales have not shown very good predictive value in practice and the literature [20]. It is evident that determining the main fall risk factors is important for fall prevention, but the prediction of fall risk through scale testing alone is often not a simple task in reality. In this study, we collected CoP trajectory data using a plantar force plate and used PCA to calculate the 95% confidence ellipse of the CoP trajectory to extract its area, long axis length, short axis length, and the angle between the long axis and the x-axis. The results found that the area and short axis length of the CoP confidence ellipse were significantly greater in diabetics than in healthy younger and older people, while the long axis length of the confidence ellipse did not differ among the three groups; healthy older people and diabetics had a greater degree of instability in the minor axis direction (AP slope plane, left or right slope plane) than healthy younger subjects.

Of the many identified risk factors for falls, the more important ones tend to be impaired balance and mobility due to age or diabetes-related declines in physiological function. Therefore, most screening tools and interventions are specifically designed to address balance, walking dysfunction, reduced responsiveness, and muscle weakness, as these factors are amenable to improvement in the elderly or diabetic population and are most likely to be positively influenced by individualized interventions [36–38]. In a systematic review by Tofthagen et al. [36], the authors summarise the existing literature on the effectiveness of plyometric and balance training interventions in people at high risk of falls and conclude that plyometric and balance training is recommended to be safe and effective in older people at high risk of falls and in the DPN population. A systematic review and meta-analysis by Thukral et al. [37], in which 18 randomized controlled trials were also pooled, showed that the use of different exercises (e.g. balance exercises, core stability exercises, Tai Chi, proprioceptive exercises, etc.) for patients with DPN had a significant positive effect on improving posture and stability. Akbari et al. [38] also conducted a meta-analysis that included 12 original studies and concluded that different types of functional body balance exercises were effective in improving static, dynamic, and functional balance parameters in patients with DPN.

In this study, the CoP trajectory data from the plantar force plate was used to assess the static standing balance of healthy older and diabetic patients, which is more accurate than many previous scales and provides a true and objective picture of the subject’s immediate standing stability. A six-degree-of-freedom perturbation platform was used to tilt the plantar force plate to a 5° slope with the human body standing statically on the slope, thereby exerting a stable and uniform horizontal shear force on the sole. The 5° angle of inclination was chosen for the following two reasons: (1) the psychological acceptance of the subjects in the pre-experiments showed that the majority of the subjects were able to complete the test at a 5° slope with relative ease, which eliminated the influence of psychological factors on the test results while ensuring the safety of the test process. (2) some pre-experimental data show that the shear force exerted on the bottom of the foot during static stance on a 5° slope is close to the horizontal shear force during the gait cycle. In this study, four different phases of stance were used to assess the stability of the subjects, namely horizontal, AP, and left–right slopes. To avoid the influence of the test environment on the mental activity of the subjects during the test, a quiet and comfortable indoor environment was chosen and the subjects were familiarised with the test environment and the test procedure before the test started. The results showed that the confidence ellipse area and the short axis length of the CoP trajectory of diabetic patients were both increased than those of healthy older and young adults, while the long axis length did not differ among the three groups. The above results show that: (1) the balance function of diabetic patients is lower than that of healthy younger and older people; (2) the increase in the amplitude of body sway of diabetic patients during static standing compared to healthy people mainly occurs in the direction of the short axis of the confidence ellipse, while there is no significant change in the amplitude of sway in the direction of the main axis. This is also reflected in the kernel density maps and circular radiograms plotted in the major axis direction of the confidence ellipse for each subject shown in Fig. 7, where diabetics have a relatively more dispersed major axis direction of sway and a relatively low kurtosis coefficient, indicating greater uncertainty in the major direction of their body sway during static standing in this group.

The following limitations of this study were noted: (1) this study was a cross-sectional observational study and subjects were not followed up relatively far into the future to observe the occurrence of outcome events such as falls, thus it was not possible to correlate the parameters associated with CoP trajectory with fall events and therefore further analysis of the relationship between the two is needed in a prospective study with a large sample; (2) no significant differences in balance function were found between healthy older people and younger people in this study. The possible reasons for this are as follows: due to the high height of the disturbance platform involved in this study (approximately 1.2 m) and to ensure the safety of the experimental procedure, the age of the healthy older group was reduced to 50–70 years, which narrowed the difference in stability between this group and the healthy younger group, and therefore failed to achieve a statistical effect under the current sample size due to a possible risk of Type II error.

## Conclusion

The balance function of the diabetic patients is lower than that of the healthy younger and older people, and the increase in body sway during static standing is mainly in the direction of the minor axis of the CoP confidence ellipse, while there is no significant change in the amplitude of sway in the major axis. To prevent falls in the diabetic and elderly population, an assessment of balance function should be carried out, and the early use of balance function exercises in people with abnormal balance functions can effectively prevent falls.

## Data Availability

The datasets used and/or analyzed during the current study are available from the corresponding author on reasonable request.

## Abbreviations

CoP: Center-of-pressure
PCA: Principal component analysis
AP: Anteroposterior
ML: Medial–lateral
DPN: Diabetic peripheral neuropathy
TTT: Transverse tibial bone transport
SD: Standard deviation
HoV: Homogeneity of variance
ANOVA: Analysis of variance
